# Validation of ring-type cuffless blood pressure (BP) monitoring device for detecting awake-asleep BP variations compared to ambulatory BP monitoring device

**DOI:** 10.1038/s41440-026-02759-6

**Published:** 2026-08-05

**Authors:** Seung-Mok Lee, Hyuktae Kwon, Belong Cho, Jin Ho Park, Sang-Hoon Na, Seung-Pyo Lee, Hae-Young Lee

**Affiliations:** 1 Division of Cardiology, Department of Internal Medicine, Seoul National University Hospital, Seoul, Republic of Korea; 2 Department of Family Medicine, Seoul National University Hospital, Seoul National University College of Medicine, Seoul, Republic of Korea; 3 Department of Internal Medicine, Seoul National University College of Medicine, Seoul, Republic of Korea

**Keywords:** Blood pressure monitoring, Ambulatory, Blood pressure monitors, Morning hypertension, Implemental hypertension, Digital hypertension

## Abstract

We evaluated the accuracy of a ring-type cuffless BP device (CART BP Pro) in detecting awake–asleep BP changes compared to conventional 24-h ambulatory BP monitoring (ABPM), following the validation protocol recommended by the European Society of Hypertension (ESH). Among 84 recruited patients, 35 were selected to satisfy ESH recommendations: 30% (*n* = 11) with systolic BP (SBP) ≥ 130 mmHg and diastolic BP (DBP) ≥ 80 mmHg and 30% (*n* = 11) with SBP < 120 mmHg and DBP < 70 mmHg. The remaining 40% (*n* = 13) were included sequentially based on recruitment order. CART BP Pro was calibrated at least one day prior to simultaneous monitoring with ABPM. Participants simultaneously wore both devices on the non-dominant arm for 24 h. Mean SBP/DBP values and awake–asleep BP changes were compared. Awake–asleep SBP/DBP changes were 15.61 ± 8.74/11.35 ± 6.22 mmHg (ABPM) and 13.87 ± 9.04/11.44 ± 8.62 mmHg (CART BP Pro). Differences in mean awake–asleep BP changes were −1.74 ± 6.47 mmHg (SBP) and 0.08 ± 5.84 mmHg (DBP). Correlation analysis showed clinically significant correlation for both SBP and DBP changes (*r* = 0.736, *p* < 0.01). Bland–Altman plots showed acceptable agreement with a mean difference and 95% limits of agreement of −1.74 (−14.42, 10.94) mmHg for SBP and 0.08 (−11.37, 11.53) mmHg for DBP. For four-category dipping classification, CART BP Pro showed moderate agreement with ABPM (weighted κ = 0.58). In conclusion, CART BP Pro showed a meaningful level of agreement with ABPM in detecting awake–asleep BP changes. As this addressed only the awake/asleep test of the ESH protocol, the device cannot yet replace conventional 24-h ABPM pending completion of the remaining validation tests.

**Figure:**
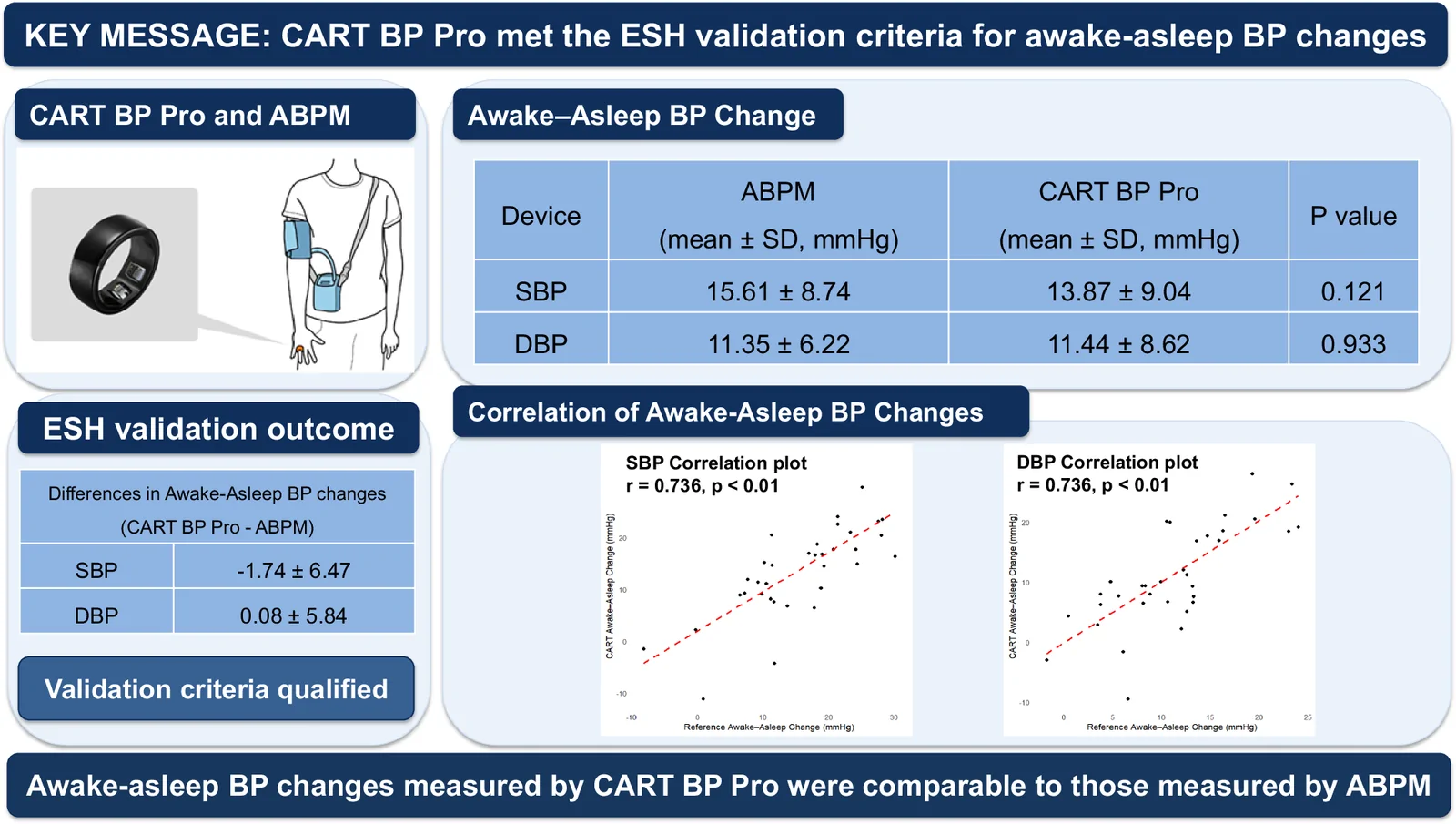

## Introduction

Blood pressure (BP) is a dynamic and essential biomarker for assessing cardiovascular risk, and elevated BP remains the most important modifiable predictor of cardiovascular morbidity and mortality [1]. Despite its critical prognostic significance, BP is a highly variable parameter influenced by behavioral, environmental, and physiological factors [2]. Consequently, repeated BP measurement over time is required to obtain an accurate assessment of an individual’s true BP burden and risk. Another prognostic indicator is diurnal variation including nocturnal dipping. Blunted nocturnal dipping is associated with an increased risk of cardiovascular events, independent of average BP levels [3, 4]. Assessment of BP variability and nocturnal dipping provides important prognostic information, allowing hypertensive patients to be stratified into groups with up to a fourfold difference in cardiovascular risk [5].

Traditionally, ambulatory blood pressure monitoring (ABPM) has been considered as the gold standard for 24-h BP monitoring. However, the current cuff-based ABPM has limitations, including discomfort from repeated cuff inflation, potential interference with sleep, and low patient adherence during prolonged monitoring periods. Moreover, only 50% of the patients replicated dipping/non-dipping phenotypes at a second ABPM [6]. In this regard, a method that achieves accuracy comparable to ABPM while avoiding the discomfort of cuff inflation could facilitate repeated assessments and thereby help to more reliably establish an individual’s dipping phenotype. To address these issues, cuffless BP monitoring technologies have emerged as a promising alternative. Cuffless devices, especially those employing photoplethysmography (PPG) or wearable platforms, offer advantages such as improved patient comfort, continuous monitoring capabilities, and better suitability for long-term BP assessment. Moreover, their potential to facilitate detection of BP variability and diurnal patterns in daily life settings may offer new clinical opportunities to optimize hypertension diagnosis and management [7–9].

Although cuffless BP devices have considerable potential for the diagnosis and management of hypertension, fundamental questions regarding their accuracy, performance, and implementation need to be carefully addressed before they can be recommended for clinical use [10, 11]. While previous studies have primarily focused on the absolute accuracy of cuffless BP devices, few have rigorously evaluated their ability to detect awake–asleep BP variation, a clinically meaningful marker of cardiovascular risk [12, 13]. Given that the dipping pattern is a critical therapeutic target, any device used in practice must reliably capture this transition. The European Society of Hypertension Working Group on BP Monitoring and Cardiovascular Variability has proposed a structured framework comprising six distinct validation tests for intermittent cuffless devices: (1) static test (absolute BP accuracy), (2) device position test (hydrostatic pressure effect robustness), (3) treatment test (BP decrease accuracy), (4) awake/asleep test (BP change accuracy), (5) exercise test (BP increase accuracy), and (6) recalibration test (cuff calibration stability over time) [14].

The ring-type cuffless BP monitoring device used in this study (CART BP Pro) is a cuff-calibrated, automated, PPG-based wearable device designed for intermittent 24-h BP monitoring. It has previously undergone static BP testing [15, 16] and a comparative study with ABPM [17], leading to its recognition as a medical device in Korea and its inclusion in the national health insurance reimbursement program for 24-h BP measurement since 2024. Building on this foundation, we aimed to extend the validation process in accordance with the ESH recommendations by conducting the awake/asleep test—the primary test for automated wearable devices—using simultaneous 24-h ABPM as the reference standard.

## Patients and methods

### Study design

**Fig. 1:**
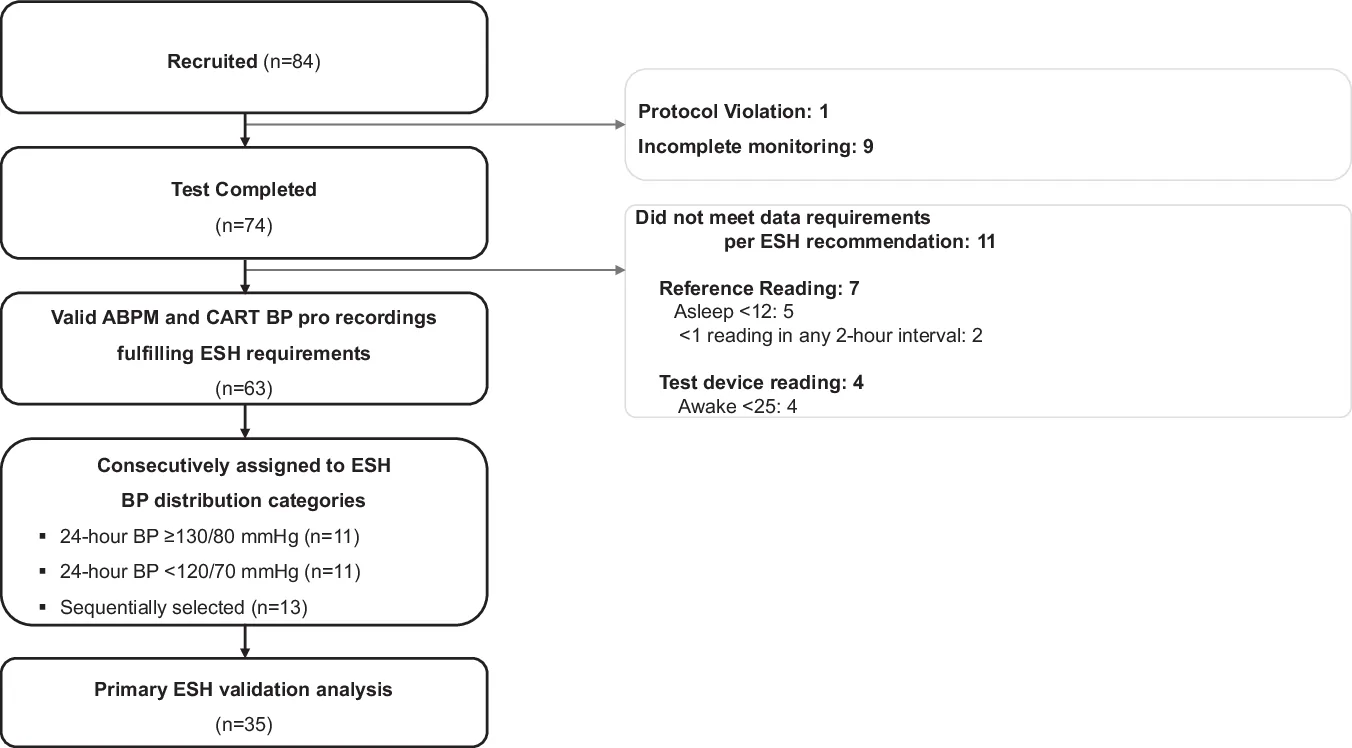
Flow diagram of participant selection and inclusion in the primary ESH validation analysis. Flow diagram showing participant enrollment, completion of monitoring, application of European Society of Hypertension (ESH) validity criteria, and selection of the final study population for the primary validation analysis. Of 84 enrolled participants, 74 completed simultaneous monitoring with ambulatory blood pressure monitoring (ABPM) and CART BP Pro. Ten participants were excluded because of protocol violation or incomplete monitoring, and an additional 11 participants were excluded because the recordings did not meet ESH minimum data requirements. Among the 63 participants with valid datasets, 35 participants were selected for the primary ESH validation analysis according to the BP distribution requirements specified in the ESH protocol. BP, blood pressure; ABPM, ambulatory blood pressure monitoring; ESH, European Society of Hypertension

Participants were recruited from individuals for whom ABPM was clinically indicated at the outpatient clinic. Exclusion criteria were body mass index ≥30 kg/m², pregnancy, significant cardiovascular disease (heart failure, valvular heart disease, or previous hospitalization for myocardial infarction), atrial fibrillation documented within the previous 6 months, end-stage renal disease, inability to provide informed consent, and inability to comply with the study protocol. During the monitoring period, participants were instructed to maintain their usual daily activities. Among 84 recruited participants, 63 fulfilled the ESH requirements for valid ABPM and CART BP Pro recordings. Exclusions were attributable to incomplete monitoring or to an insufficient number of valid readings caused by motion artifacts, which failed to satisfy the ESH minimum reading requirements. To meet the BP distribution mandated by the ESH protocol—at least 30% of participants with systolic BP (SBP) ≥ 130 mmHg and diastolic BP (DBP) ≥ 80 mmHg, and at least 30% with SBP < 120 mmHg and DBP < 70 mmHg—participants were enrolled consecutively in their order of recruitment and assigned to the corresponding BP category until each category was filled, with the remaining participants enrolled sequentially to complete the required sample of 35 (Fig. 1). Participants were therefore not selected on the basis of the magnitude of any discrepancy between the reference and test measurements, and individuals at the extremes of the BP range were neither preferentially included nor excluded.

As illustrated in the graphical abstract, the test device (CART BP Pro; Sky Labs Inc., Seongnam, Korea) was worn on the finger of non-dominant wrist to minimize motion artifacts. The reference device, a standard ABPM device (TM-2440, A&D Medical, Tokyo, Japan), was worn on the same (non-dominant) arm. Participants wore both devices concurrently for ~24 h and were allowed to wear the device on any finger except the thumb according to comfort and practical considerations. ABPM device recorded BP every 20 min. CART BP Pro continuously collected PPG signals for 15 s at 1-min intervals. All validated reference data were included in the analysis regardless of the availability or quality of the corresponding CART BP Pro measurements.

This study was approved by the Institutional Review Board of Seoul National University Hospital (E-2305-064-1431), and all participants provided written informed consent. The study was registered at ClinicalTrials.gov (NCT06084065) and conducted in accordance with the Declaration of Helsinki (2013).

### Data preprocessing

Data preprocessing was previously reported [15]. Briefly, continuous PPG signals were processed using the device’s inherent algorithm after signal quality assessment. Segments affected by motion artifacts or cuff inflation were excluded, whereas all remaining valid signals were continuously used for BP estimation. Valid BP estimates obtained throughout the monitoring period were then aggregated within the predefined awake and asleep periods to generate representative BP values for comparison with ABPM. Thus, BP estimates were derived from the continuous cuffless monitoring data, excluding only artifact-affected segments, rather than being restricted to measurements linked to specific ABPM time points.

### Calibration method and data analysis

CART BP Pro, which needs cuff-based calibration, was calibrated at least one day prior to simultaneous BP monitoring with the ABPM device (Supplementary Table 1), as recommended by the ESH for the awake/asleep validation of cuff-calibrated wearable devices [14]. The calibration procedure followed the manufacturer-recommended protocol and was not modified for the purpose of this study. The same ABPM device was used during calibration and subsequent 24-h monitoring. Participants were seated in a resting position during calibration with the arm supported at heart level. Calibration was conducted using an ABPM device via a reference–test–reference–test–reference (R-T-R-T-R) sequence. The average of the first and second reference readings (R1 and R2) were paired with the first test measurement (T1), and the average of the second and third reference readings (R2 and R3) were paired with the second test measurement (T2). A minimum 1-minute interval was maintained between measurements to allow vascular recovery and ensure high-quality PPG acquisition.

Sensitivity analyses were additionally performed in the full valid cohort (*n* = 63) and in the remaining valid participants not included in the ESH validation subset (*n* = 28).

### Statistical analysis

Test device performance was evaluated against the ESH awake/asleep validation standards: (1) mean difference between test and reference SBP/DBP changes ≤5 mmHg, (2) standard deviation (SD) of the differences ≤8 mmHg, and (3) correlation coefficient (r) ≥0.70 for both SBP and DBP. SBP and DBP values recorded during daytime and nighttime periods by both devices were analyzed. Mean SBP and DBP were calculated for 24-h, daytime, and nighttime periods. Awake and asleep periods were defined using participant-reported sleep diaries. Accuracy was assessed via mean error (test minus reference) and corresponding SD. Pearson correlation coefficients were used to evaluate linear association. Agreement between devices was assessed using Bland–Altman plots. For dipping status, agreement was evaluated by detailed classification into four categories (cut-offs of 80%, 90%, and 100%). Agreement between devices was quantified using Cohen’s κ, and potential systematic bias was examined with Bowker’s test. All analyses were performed in R (version 4.4.3), with a two-sided *p*-value < 0.05 considered statistically significant.

## Results

### Patient’s characteristics

Thirty-five participants were consecutively selected from 84 initially recruited patients to ensure compliance with the ESH recommendations: 30% (*n* = 11) with SBP ≥ 130 mmHg and DBP ≥ 80 mmHg, and 30% (*n* = 11) with SBP < 120 mmHg and DBP < 70 mmHg, with the remaining 40% (*n* = 13) included sequentially based on recruitment order (Fig. 1). Exclusions were due to the following: protocol violation (*n* = 1), incomplete monitoring (*n* = 9), insufficient reference device readings (*n* = 7), and insufficient test device readings (*n* = 4). Baseline demographic characteristics of the 35 participants included in the final analysis are summarized in Table 1. The average age, height, weight, and BMI of the participants were 53.5 ± 11.7 years, 166.8 ± 9.4 cm, 67.7 ± 14.8 kg, and 24.2 ± 4.2 kg/m², respectively. Seventeen participants (48.6%) were female. The mean office SBP/DBP were 119.7 ± 14.9 mmHg and 79.0 ± 9.5 mmHg, respectively. Based on ABPM, 11 participants met the criteria for 24-h BP ≥ 130/80 mmHg, while another 11 participants had 24-h BP < 120/70 mmHg. Daytime BP ≥ 135/85 mmHg and nighttime BP ≥ 120/70 mmHg were observed in 7 and 9 participants, respectively. A total of 13 participants had hypertension, among whom 6 were receiving two or more classes of antihypertensive agents. Four participants had diabetes mellitus.

**Table 1:** Baseline characteristics of participants included in the primary ESH validation analysis (*n* = 35)

Variable	Value (mean ± SD or *n* (%))
Age (yo)	53.5 ± 11.7
Height (cm)	166.8 ± 9.4
Weight (kg)	67.7 ± 14.8
BMI (kg/m^2^)	24.2 ± 4.2
Female	17 (48.6)
Office SBP (mmHg)	119.7 ± 14.9
Office DBP (mmHg)	79.0 ± 9.5
24 h BP ≥ 130/80 mmHg	11 (31.4)
24 h BP < 120/70 mmHg	11 (31.4)
Daytime BP ≥ 135/85 mmHg	7 (20)
Nighttime BP ≥ 120/70 mmHg	9 (25.7)
Hypertension	13 (37.1)
Antihypertensive ≥2	6 (17.1)
Diabetes	4 (11.4)

Data are presented as mean ± standard deviation (SD) or number (%), as appropriate. Participants were selected according to the blood pressure distribution requirements specified in the European Society of Hypertension (ESH) validation protocol

*BMI* body mass index, *BP* blood pressure, *SBP* systolic blood pressure, *DBP* diastolic blood pressure

### Comparison of mean BP values between two devices

The mean SBP and DBP values and their differences measured by the CART BP Pro and the ABPM device during the 24-h, awake, and asleep periods are presented in Table 2. Across all periods, mean differences of SBP ranged from 0.13 mmHg to 2.16 mmHg for SBP and from 1.23 mmHg to 1.78 mmHg for DBP. The standard deviations were between 7.21 mmHg and 7.87 mmHg for SBP and 4.44 mmHg and 6.36 mmHg for DBP, respectively. *p*-values for each time period were >0.05 except for awake DBP, which also suggests the ability of CART BP Pro to detect BP accurately.

**Table 2:** Comparison of 24-h, awake, and asleep blood pressure measurements between CART BP Pro and ABPM

Measure		ABPM	CART BP pro	Differences (Mean ± SD)	*p* value
SBP	24 h	120.9 ± 17.1 mmHg	121.1 ± 16.2 mmHg	0.13 ± 7.21	0.916
	Awake	125.4 ± 17.2 mmHg	125.8 ± 15.9 mmHg	0.42 ± 7.44	0.741
	Asleep	109.8 ± 17.6 mmHg	111.9 ± 17.2 mmHg	2.16 ± 7.87	0.114
DBP	24 h	76.9 ± 9.9 mmHg	78.1 ± 9.4 mmHg	1.23 ± 4.54	0.117
	Awake	80.2 ± 10.5 mmHg	81.9 ± 9.8 mmHg	1.78 ± 4.44	0.024
	Asleep	68.8 ± 9.1 mmHg	70.5 ± 10.6 mmHg	1.69 ± 6.36	0.125

Values are presented as mean ± standard deviation (SD). Differences were calculated as CART BP Pro minus ABPM. *P* values were obtained using paired comparisons between devices

*ABPM* ambulatory blood pressure monitoring, *SBP* systolic blood pressure, *DBP* diastolic blood pressure

### Awake–asleep BP change within each device

Both devices showed a typical nocturnal dipping pattern, with lower BP during the asleep period than the awake period. The mean changes in SBP and DBP (awake–asleep) were 15.61 mmHg ± 8.74 mmHg and 11.35 ± 6.22 mmHg for ABPM, and 13.87 ± 9.04 mmHg and 11.44 ± 8.62 mmHg for CART BP Pro, respectively (Table 3). Differences in awake–asleep BP changes between CART BP Pro and ABPM were −1.74 ± 6.47 mmHg for SBP, and 0.08 ± 5.84 mmHg for DBP, suggesting an acceptable difference between the two devices in terms of detecting awake–asleep BP changes according to the recent ESH recommendations. *p*-values were 0.121 for SBP and 0.933 for DBP. This result demonstrates the consistency of CART BP Pro with ABPM to detect awake–asleep BP change in terms of nocturnal dipping pattern and magnitude.

**Table 3:** Comparison of awake–asleep blood pressure changes measured by CART BP Pro and ABPM

Device	ABPM (mean ± SD)	CART BP Pro (mean ± SD)	Differences (mean ± SD)	*p* value
SBP	15.61 ± 8.74 mmHg	13.87 ± 9.04 mmHg	−1.74 ± 6.47	0.121
DBP	11.35 ± 6.22 mmHg	11.44 ± 8.62 mmHg	0.08 ± 5.84	0.933

Awake–asleep blood pressure (BP) change was calculated as awake BP minus asleep BP. Values are presented as mean ± standard deviation (SD). Differences were calculated as CART BP Pro minus ABPM. *P* values were obtained from paired comparisons between devices

*ABPM* ambulatory blood pressure monitoring, *SBP* systolic blood pressure, *DBP* diastolic blood pressure

### Correlation and agreement of BP change between devices

**Fig. 2:**
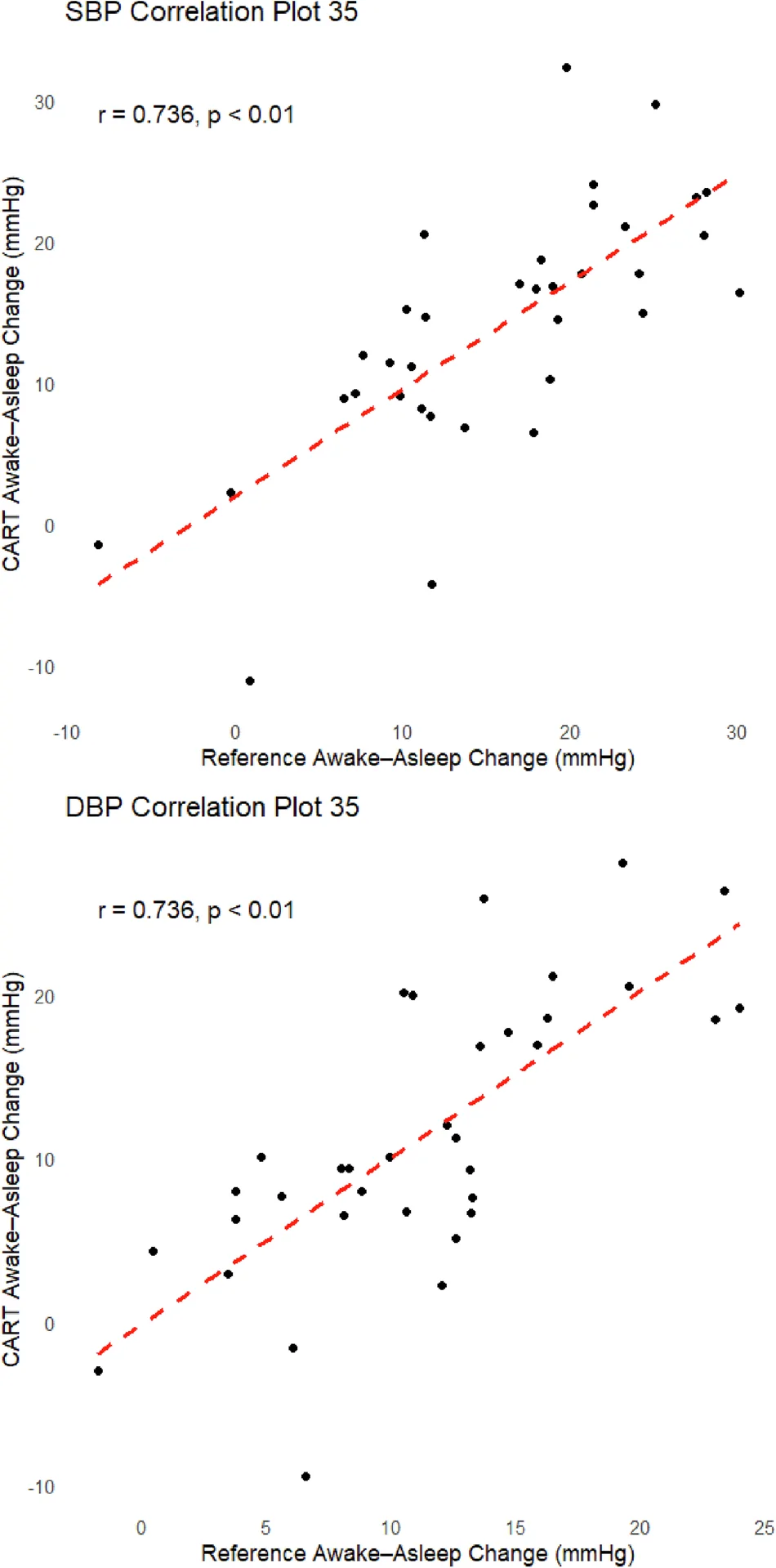
Correlation between awake–asleep blood pressure changes measured by CART BP Pro and ABPM. Scatter plots showing the relationship between awake–asleep blood pressure (BP) changes measured by CART BP Pro and ambulatory blood pressure monitoring (ABPM). The upper scatter plot shows systolic BP (SBP) changes and the lower scatter plot shows diastolic BP (DBP) changes. Awake–asleep BP change was calculated as awake BP minus asleep BP. Pearson correlation coefficients were 0.736 for both SBP and DBP (*p* < 0.01 for each). The dashed red line represents the linear regression line. ABPM was used as the reference method

Figure 2 shows the correlation plots of awake–asleep changes between the two devices. Awake–asleep changes in SBP and DBP showed moderate to strong correlation between the devices. Correlation coefficients for SBP and DBP were both 0.736 with *p*-values < 0.01, suggesting significant correlation between awake–asleep changes based on BP measurements by the two devices.

**Fig. 3:**
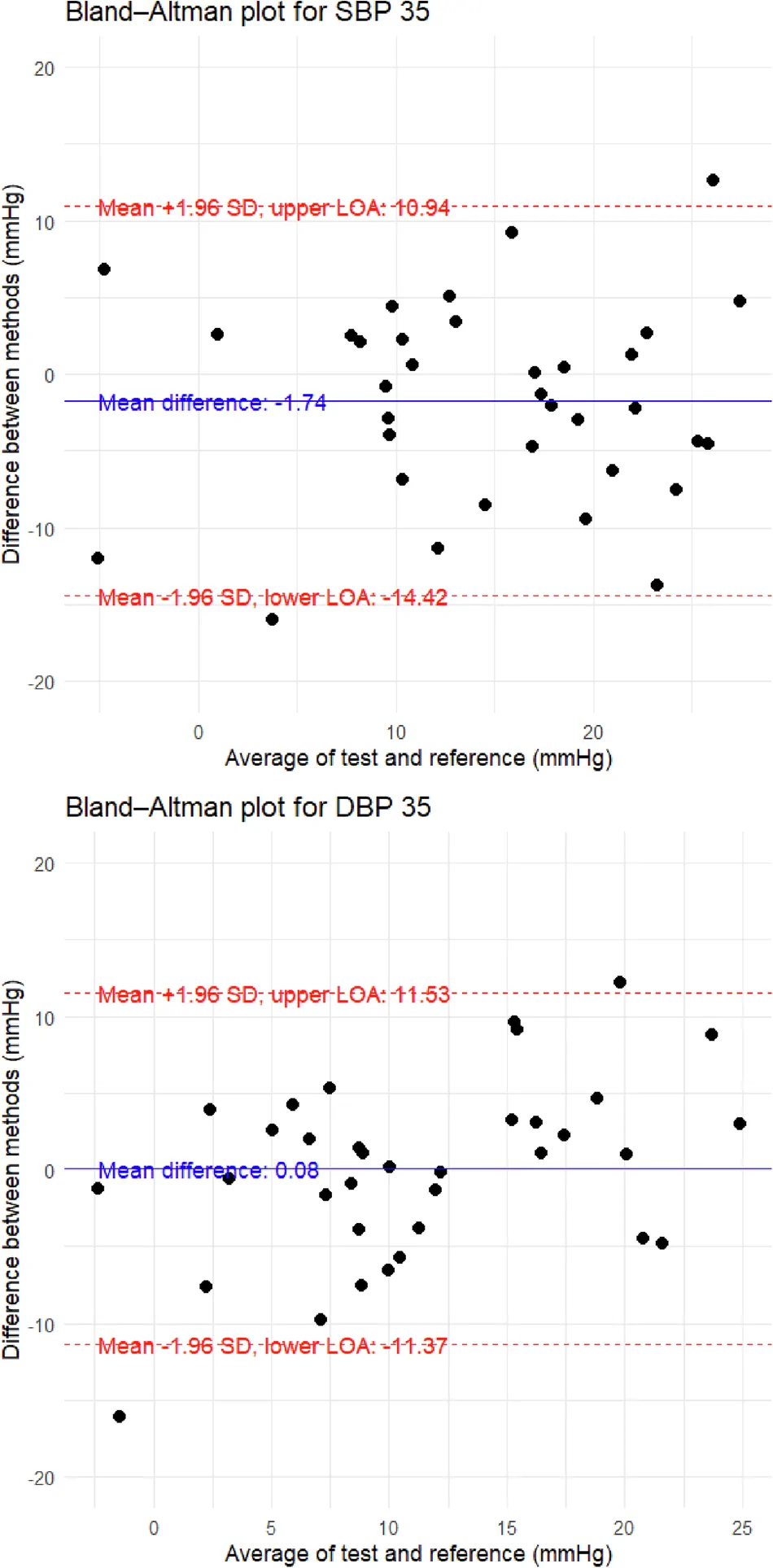
Bland–Altman analysis of agreement for awake–asleep blood pressure changes between CART BP Pro and ABPM. Bland–Altman plots comparing awake–asleep blood pressure (BP) changes measured by CART BP Pro and ambulatory blood pressure monitoring (ABPM). The upper panel shows systolic BP (SBP) changes and the lower panel shows diastolic BP (DBP) changes. Differences were calculated as CART BP Pro minus ABPM. The solid blue line indicates the mean difference, and the dashed red lines indicate the 95% limits of agreement (mean difference ±1.96 SD). The x-axis represents the average of the CART BP Pro and the ABPM awake–asleep BP changes

Bland–Altman plots (shown in Fig. 3) revealed a mean difference of −1.74 mmHg for awake–asleep SBP changes with 95% limits of agreement ranging from −14.42 mmHg to 10.94 mmHg. The mean difference for awake–asleep DBP changes was 0.08 mmHg, and 95% limits of agreement were from −11.37 mmHg to 11.53 mmHg. No significant systematic bias was observed.

Sensitivity analyses performed in the full valid cohort (*n* = 63) and in participants not included in the ESH validation subset (*n* = 28) demonstrated similar mean differences, correlation coefficients, and pass status according to the ESH awake/asleep test criteria (Supplementary Table 2, Supplementary Figs. 1, 2), supporting the robustness of the primary findings.

### Agreement in dipper classification between devices

The four-category dipping classification (extreme dipper, dipper, non-dipper, and reverse dipper) showed moderate agreement between CART BP Pro and ABPM according to the quadratic weighted κ. The quadratic weighted κ was 0.58 (95% CI, 0.35–0.73; *p* < 0.001), suggesting that the level of concordance significantly exceeded what would be expected by chance alone (Table 4).

**Table 4:** Agreement between CART BP Pro and ABPM for four-category nocturnal dipping classification

Device		CART BP Pro			
		ED	D	ND	RD
ABPM	ED	1	3	0	0
	D	2	12	8	0
	ND	0	1	4	2
	RD	0	0	1	1

Cross-tabulation of nocturnal dipping categories determined by CART BP Pro and ambulatory blood pressure monitoring (ABPM). Dipping categories were defined according to the percentage decline in nocturnal systolic blood pressure: extreme dipper (≥20%), dipper (10–<20%), non-dipper (0– < 10%), and reverse dipper (<0%). Agreement between devices was assessed using quadratic weighted Cohen’s kappa and Bowker’s test of symmetry

*ED* extreme dipper, *D* dipper, *ND* non-dipper, *RD* reverse dipper

## Discussion

In this validation study, we demonstrated that a ring-type cuffless BP monitoring device, CART BP Pro, showed acceptable agreement with conventional 24-h ABPM in detecting awake–asleep BP variations. Across the 24-h, daytime, and nighttime periods, mean SBP and DBP values measured by CART BP Pro were comparable to those measured by ABPM and remained within acceptable limits of difference, with awake DBP being the only parameter showing a statistically significant difference. Correlation analysis and Bland–Altman plots support the device’s capability to track dynamic BP changes over the circadian cycle. In terms of dipper classification, CART BP Pro showed moderate agreement with ABPM. To the best of our knowledge, this is the first study to follow the ESH validation protocol, specifically conducting the awake/asleep test after completion of the static BP test, thereby providing a rigorous evaluation of diurnal BP variation detection in a cuffless device.

Compared with previous validation studies of cuffless BP devices, our study uniquely addressed awake–asleep BP transitions, a critical parameter in cardiovascular risk stratification. Many earlier validation studies have focused primarily on point accuracy or daytime BP measurements [13]. Participants were recruited across a broad BP spectrum to ensure balanced BP distribution fulfilling the criteria recommended by the ESH, and results demonstrated robust performance regardless of baseline BP level. These findings are aligned with evolving hypertension management strategies that emphasize both average BP and BP variability [2].

Despite the promising results of our study, it is important to acknowledge the ongoing scientific concerns surrounding cuffless BP monitoring devices. Technical limitations remain, including dependence on calibration stability, potential susceptibility to motion artifacts, and variability in accuracy across skin tones, physiological states, and environmental conditions [12, 18]. Furthermore, large-scale longitudinal studies demonstrating the prognostic relevance of cuffless-derived BP parameters are still lacking, and standardized international protocols for device validation are only beginning to be implemented. Nevertheless, public perception and acceptance of cuffless BP technologies appear to be markedly more open-minded than the scientific community’s cautious stance, driven by the comfort, convenience, and seamless integration of such devices into daily life [19, 20]. A major milestone was the recent FDA clearance of the Aktiia device—the first cuffless BP monitor to receive such regulatory approval—underscoring a growing recognition of their potential for clinical use. These trends suggest that continued technological advancement, combined with rigorous adherence to standardized validation frameworks such as the ESH protocol, will accelerate the clinical adoption and integration of cuffless BP monitoring into hypertension diagnosis and management.

In this study, we demonstrated that a ring-type cuffless BP device, CART BP Pro, shows promising accuracy in assessing diurnal BP variation, including the detection of dipping status. These findings are in line with the evolving understanding of BPV as an independent predictor of cardiovascular events, beyond average BP levels [5]. For the evaluation of BPV, repeated BP assessment in real-life contexts is essential to capture this dynamic variability. While ABPM remains the reference standard, its practical limitations such as poor tolerability and low adherence in long-term use may hinder its utility in long-term, repetitive monitoring. In contrast, cuffless devices offer continuous, comfortable, and user-friendly monitoring, providing a critical opportunity for clinical implementation of BPV assessments—an important but underutilized metric due to the measurement limitations of ABPM [21]. Recent guidelines from both the ESH and American expert consensus acknowledge the growing role of cuffless BP monitoring but emphasize the need for rigorous validation [9]. Our findings support the integration of cuffless BP monitoring as a complementary method, particularly for evaluating circadian patterns and BPV. Nevertheless, before wide adoption, standardization of validation protocols and demonstration of prognostic relevance are required, as current technologies vary significantly in methodology, calibration needs, and accuracy.

While CART BP Pro demonstrated good agreement with ABPM for awake–asleep BP changes at the population level, the relatively wide limits of agreement observed in Bland–Altman analyses suggest that individual-level differences may be clinically relevant in some cases. This may partly account for the moderate agreement observed for dipping classification. In addition, the known limited reproducibility of dipping phenotypes even with repeated ABPM assessments should be considered when interpreting these findings. Accordingly, although CART BP may be useful for tracking nocturnal BP patterns, caution is warranted when applying dipping classifications at the individual level, particularly when such classifications are used to guide clinical decision-making.

An additional consideration is the completeness of valid recordings during 24-h monitoring. In the present study, four participants were excluded because of insufficient valid CART BP Pro recordings, whereas seven participants were excluded because of insufficient valid reference ABPM recordings. These findings suggest that data loss was not unique to the cuffless device but reflects, at least in part, the practical challenges of obtaining adequate 24-h ambulatory BP measurements. Nevertheless, because this study was conducted under a controlled validation protocol, the recording performance and usability of CART BP Pro in less controlled real-world settings remain to be established. Further studies are needed to evaluate device performance, user adherence, and recording completeness during routine daily activities.

This study also has some limitations. First, the 35 participants analyzed were not enrolled at random but were recruited consecutively to satisfy the BP distribution mandated by the ESH validation protocol, which requires at least 30% of participants with 24-h SBP ≥ 130 mmHg and DBP ≥ 80 mmHg and at least 30% with SBP < 120 mmHg and DBP < 70 mmHg. Participants meeting each criterion were enrolled in their order of recruitment until the required quota (11 participants per category) was reached, and the remaining participants were added sequentially. Crucially, this protocol-driven procedure does not constitute selection on the basis of device agreement: individuals were neither preferentially included nor excluded according to the magnitude of any discrepancy between the reference and test measurements, nor according to their position at the extremes of the BP range. To confirm that this recruitment scheme did not bias our results, we performed sensitivity analyses in the full valid cohort (*n* = 63) and in the participants not assigned to the predefined BP categories; both yielded agreement and correlation comparable to the primary analysis, indicating that the findings were robust to the enrollment procedure. Second, the relatively small sample size may restrict the power of subgroup analyses. Third, direct measures of arterial stiffness or subclinical atherosclerotic burden were not available. Because vascular properties may influence the performance of cuffless BP monitoring devices, future studies should evaluate device performance across a broader spectrum of arterial stiffness and atherosclerotic disease. Fourth, the reproducibility of dipping and non-dipping phenotypes is known to be limited even with repeated ABPM assessments [6]. As a result, concordance between CART BP Pro and ABPM may be influenced not only by device performance but also by the inherent variability of dipping status itself, and ABPM cannot be regarded as an error-free reference for an individual’s “true” dipping phenotype. Nevertheless, because ABPM is currently regarded as the reference standard for evaluating nocturnal dipping patterns, it was used as the comparator in this study. As noted in the introduction, improving the reproducibility of dipping classification ultimately requires repeated nocturnal assessments, which are difficult to obtain with conventional ABPM because cuff inflation disturbs sleep and limits patient acceptance of repeated examinations. Given its unobtrusive and discomfort-free nature, cuffless monitoring with accuracy comparable to ABPM may offer a practical advantage in this setting, and further studies evaluating whether serial cuffless measurements can establish a more stable estimate of an individual’s habitual dipping phenotype are warranted. Lastly, all measurements were obtained in a controlled research setting, and real-world performance in ambulatory or physically active conditions remains to be evaluated. Moreover, although the overall correlation between the two devices was robust, a small but statistically significant difference was observed in awake DBP values. Given the limited sample size, this statistical significance may reflect a type I error. This finding highlights the need for larger-scale studies to confirm whether such discrepancies persist and to ensure the consistency of device agreement across diverse populations and clinical scenarios.

Despite these limitations, our findings provide meaningful insight into the clinical potential of a ring-type cuffless BP monitor for ambulatory use. The ability to reliably detect awake–asleep BP variations without a cuff opens possibilities for more comfortable and accessible 24-h BP monitoring without deteriorating sleep quality. The present study assessed only the awake/asleep test, which is the principal test of the ESH validation framework for cuff-calibrated wearable cuffless devices but is one of six required components [14]. The static test has previously been completed [16], and the device position and exercise tests have been completed by independent investigators and are currently under peer review (personal communication); the device position test is of particular relevance for a finger-based device. The recalibration (stability over time) test is ongoing, and the treatment-induced change test is being evaluated through incorporation of the device as a secondary endpoint in several phase IV pharmacological trials. Of note, CART BP Pro received approval from the Ministry of Food and Drug Safety (MFDS) of the Republic of Korea for BP measurement in 2023 and has been reimbursed for 24-h ABPM in Korea since June 2024. Therefore, it is an established measurement tool rather than a prototype. Completion of these remaining steps will be necessary before the device can be regarded as fully validated for clinical use as a 24-h BP monitoring system.

## Conclusion

In this study, CART BP Pro showed promising performance in detecting awake–asleep BP changes and nocturnal dipping patterns compared with ABPM. As this addressed only the awake/asleep test of the ESH protocol, the device cannot yet be regarded as a fully validated 24-h BP monitor or a substitute for conventional ABPM, pending completion of the remaining validation tests.

## Supplementary information

Supplementary Table 1

Supplementary Table 2

Supplementary Figure 1

Supplementary Figure 2
