# Supplementary material for: Validation of ring-type cuffless blood pressure (BP) monitoring device for detecting awake-asleep BP variations compared to ambulatory BP monitoring device
Source: Hypertens Res. 2026 Aug 5;49(10):2817–25. doi: 10.1038/s41440-026-02759-6 (PMC13634909; doi:10.1038/s41440-026-02759-6)
Supplement: Supplementary file 1 [file 41440_2026_2759_MOESM1_ESM.docx]

**Supplementary Table 1. Distribution of Calibration-to-Monitoring Time Intervals for CART BP Pro**

| **Group** | **n** | **Median [IQR], hours** | **Min-Max, hours** |
| --- | --- | --- | --- |
| **Full valid cohort** | 63 | 24.19 [24.00-24.43] | 21.25-73.87 |
| **ESH validation subset** | 35 | 24.11 [23.99-24.36] | 21.25-73.87 |
| **Non-selected participants** | 28 | 24.24 [24.03-24.49] | 23.65-71.66 |

Legends: Distribution of calibration-to-monitoring time interval for CART BP Pro. Data are presented as median [interquartile range (IQR)] and minimum–maximum values. The full valid cohort included all participants with valid paired recordings (n=63). The ESH validation subset consisted of participants included in the primary validation analysis according to the European Society of Hypertension (ESH) protocol (n=35), whereas the non-ESH subset comprised valid participants not included in the ESH validation subset (n=28).
