# Supplementary material for: Validation of ring-type cuffless blood pressure (BP) monitoring device for detecting awake-asleep BP variations compared to ambulatory BP monitoring device
Source: Hypertens Res. 2026 Aug 5;49(10):2817–25. doi: 10.1038/s41440-026-02759-6 (PMC13634909; doi:10.1038/s41440-026-02759-6)
Supplement: Supplementary file 2 [file 41440_2026_2759_MOESM2_ESM.docx]

**Supplementary Table 2. Sensitivity analyses of awake–asleep BP changes measured by ABPM and CART BP Pro in the full valid cohort and non-selected participants.**

| **Group** | **Device** | **ABPM** | **CART BP Pro** | **Differences** | ***p-*value** |
| --- | --- | --- | --- | --- | --- |
| **Full valid cohort** | **SBP** | 13.68 ± 9.99 | 13.61 ± 9.71 | -0.07 ± 7.14 | 0.938 |
|  | **DBP** | 10.39 ± 6.81 | 11.30 ± 8.7 | 0.91 ± 5.76 | 0.216 |
| **Non-selected participants** | **SBP** | 11.26 ± 11.05 | 13.27 ± 10.65 | 2.01 ± 7.49 | 0.162 |
|  | **DBP** | 9.19 ± 7.43 | 11.12 ± 8.95 | 1.93 ± 5.58 | 0.078 |

Legends: ABPM means ambulatory blood pressure monitoring. BP Data are presented as mean ± standard deviation (SD). Awake–asleep BP change was calculated as the difference between mean awake BP and mean asleep BP. Differences were calculated as CART BP Pro minus ABPM values. All BP values are expressed in mmHg. Non-selected participants refer to participants who were excluded from the final validation cohort because of the blood pressure distribution requirements specified in the study protocol. P-values were obtained using paired t-tests comparing awake–asleep BP changes between devices.
