# Supplementary material for: Validation of ring-type cuffless blood pressure (BP) monitoring device for detecting awake-asleep BP variations compared to ambulatory BP monitoring device
Source: Hypertens Res. 2026 Aug 5;49(10):2817–25. doi: 10.1038/s41440-026-02759-6 (PMC13634909; doi:10.1038/s41440-026-02759-6)
Supplement: Supplementary file 3 [file 41440_2026_2759_MOESM3_ESM.docx]

**Supplementary Figure 1. Correlation between awake–asleep BP changes measured by ABPM and CART BP Pro in the full valid cohort and non-selected participants.**

Legends: Scatter plots showing the correlation between awake–asleep systolic blood pressure (SBP) changes (left panels) and diastolic blood pressure (DBP) changes (right panels) measured by ABPM (reference) and CART BP Pro. The upper panels represent the full valid cohort (n = 63), and the lower panels represent the non-selected participants (n = 28). Awake–asleep BP change was calculated as the difference between mean awake BP and mean asleep BP. The dashed red line indicates the line of identity. Pearson correlation coefficients (r) and corresponding p-values are shown in each panel. All blood pressure values are expressed in mmHg.
