# Supplementary material for: Validation of ring-type cuffless blood pressure (BP) monitoring device for detecting awake-asleep BP variations compared to ambulatory BP monitoring device
Source: Hypertens Res. 2026 Aug 5;49(10):2817–25. doi: 10.1038/s41440-026-02759-6 (PMC13634909; doi:10.1038/s41440-026-02759-6)
Supplement: Supplementary file 4 [file 41440_2026_2759_MOESM4_ESM.docx]

**Supplementary Figure 2. Bland–Altman analysis of awake–asleep BP changes measured by ABPM and CART BP Pro in the full valid cohort and non-selected participants.**

Legends: Bland–Altman plots comparing awake–asleep systolic blood pressure (SBP) changes (left panels) and diastolic blood pressure (DBP) changes (right panels) measured by CART BP Pro and ABPM. The upper panels represent the full valid cohort (n = 63), and the lower panels represent the non-selected participants (n = 28). Awake–asleep BP change was calculated as the difference between mean awake BP and mean asleep BP. Differences were calculated as CART BP Pro minus ABPM values and plotted against the average of the two measurements. The solid blue line represents the mean difference (bias), and the dashed red lines represent the 95% limits of agreement (mean difference ± 1.96 standard deviations). All BP values are expressed in mmHg.
